# Once-Weekly Somapacitan as an Alternative Management of Growth Hormone Deficiency in Prepubertal Children: A Systematic Review and Meta-Analysis of Randomized Controlled Trial

**DOI:** 10.3390/children11020227

**Published:** 2024-02-09

**Authors:** Ghina Tsurayya, Cut Alifiya Nazhifah, Muhammad Rahmat Pirwanja, Putri Oktaviani Zulfa, Muhammad Raihan Ramadhan Tatroman, Fajar Fakri, Muhammad Iqhrammullah

**Affiliations:** 1 Medical Research Unit, School of Medicine, Universitas Syiah Kuala, Banda Aceh 23111, Indonesia; tsurayyaghina464@gmail.com (G.T.); cutalifiyaaa@gmail.com (C.A.N.); rahmadpirwanja@gmail.com (M.R.P.); zulfaputrioktaviani@gmail.com (P.O.Z.); raihantatroman@gmail.com (M.R.R.T.); 2 Department of Pharmacy, Faculty of Mathematics and Natural Sciences, Universitas Syiah Kuala, Banda Aceh 23111, Indonesia; 3 Postgraduate Program of Public Health, Universitas Muhammadiyah Aceh, Banda Aceh 23123, Indonesia; m.iqhram@oia.usk.ac.id

**Keywords:** growth hormone, Somapacitan, GHD, insulin-like growth factor-1, Norditropin

## Abstract

Growth hormone treatment has effectively restored normal growth in children with growth hormone deficiency (GHD); however, it poses challenges in compliance with a daily growth hormone injection regimen, leading to low adherence and persistence rates. Once-weekly Somapacitan is a potential alternative for treating children with GHD. This study aimed to evaluate the efficacy, safety, and adherence of once-weekly subcutaneous Somapacitan compared to daily growth hormone injection in prepubertal children with GHD. A search for the published records was carried out on 17 October 2023 utilizing the searching feature available on PubMed, Embase, and Scopus. Primary study outcomes included (1) efficacy, measured by height velocity (HV), standard deviation score (SDs), height SDs, insulin-like growth factor-SDs (IGF-I SDs), and bone age vs. chronological age ratio (BA vs. CA); (2) safety, assessed through adverse events and injection site reactions; and (3) adherence, determined by the percentage of the sample completing treatments. Secondary outcomes evaluated disease burden scores, divided into three subgroup domains: emotional well-being, physical functional, and social well-being scores. We retrieved 6 studies that were eligible for the systematic review (417 versus 186 for intervention and control, respectively). Only 2 of the total included studies were eligible for pooled analysis (175 versus 82 for intervention and control, respectively). The efficacy profile of Somapacitan was similar to daily growth hormones, indicated by HV (mean difference (MD = 0.04; *p* = 0.96), HV SDs (MD = −0.71; *p* = 0.09), height SDs (MD = 0.11; *p* = 0.69), IGF-I SDs (MD = 0.06; *p* = 0.70), and CA vs. BA (MD = 0.67; *p* = 0.70)), demonstrated similar and non-inferior outcomes. Treatment adherence is 3 times higher in the Somapacitan group as compared to control (OR = 3.02; *p* = 0.03) with adherence rates reaching 95% and 88% for Somapacitan and Norditropin^®^, respectively. The disease burden measurement is similar in Somapacitan and daily growth hormones (MD = −0.62; *p* = 0.83), as indicated by the Growth Hormone Deficiency–Child Impact Measure. In almost all outcomes, the level of confidence is strong. The confidence level in the data is generally strong, but for CA vs. BA and the subgroup of severe adverse events with heterogeneity >50%, the confidence level is moderate. Although the efficacy and safety profiles of Somapacitan were found to be similar to those of daily growth hormones, a reduced frequency of once-weekly Somapacitan injections led to increased adherence. PROSPERO registration: CRD42023473209.

## 1. Introduction

Growth hormone deficiency (GHD) is a rare condition affecting approximately 1 in 3500–10,000 children, resulting in limited growth hormone production and impaired growth, muscle mass, bone, and fat distribution [1,2]. Children with GHD often have slower growth, a compact build, shorter stature, and may appear younger than peers of the same age and gender [2,3]. Hence, GHD can adversely impact affected children’s quality of life and emotional well-being, leading to decreased functional capacity in adulthood [4].

Growth hormone treatment has effectively restored normal growth in children with GHD, showing no significant safety concerns [5,6]. However, some studies highlighted challenges in compliance with a daily growth hormone injection regimen, leading to low adherence and persistence rates [7,8]. The need for daily injections has been burdensome for both children and caregivers, potentially affecting growth velocity [8,9]. Moreover, fear of needles and the frequency of injections pose obstacles to treatment in pediatric patients [10]. Therefore, alternative therapy is needed to address these challenges.

Somapacitan, one of the long-acting growth hormone (LAGH) types, is a novel and reversible derivative of human growth hormones that possesses albumin-binding properties, offering a long-acting alternative for treating GHD in children [11]. Designed to bind to endogenous albumin reversibly, it results in delayed elimination, a prolonged half-life, and an extended duration of action. On 28 April 2023, Sogroya^®^ (Somapacitan by Novo Nordisk Inc., Plainsboro, NJ, USA, 08536), receiving approval from the U.S. Food and Drug Administration (FDA), became the first and only once-weekly growth hormone treatment for children and adults with GHD aged 2.5 years and older [12].

A previous systematic review and meta-analysis that assessed LAGHs suggest no difference in efficacy and safety between LAGHs and daily GHs. Nonetheless, it did not evaluate the efficacy of Somapacitan due to limited studies [13]. Additionally, no other previous studies have reviewed the efficacy, safety, and adherence of Somapacitan to ameliorate GHD in children. To address this gap, the present study aims to assess the efficacy, safety, and adherence of once-weekly subcutaneous administration of Somapacitan compared to the conventional daily administration of growth hormones in the treatment of prepubertal children with GHD. To the best of our knowledge, the present study is the first systematic review evaluating Somapacitan as a potential alternative for GHD treatment in children.

## 2. Methods

### 2.1. Protocols and Registration

Protocols for this present systematic review were designed in accordance with the Preferred Reporting Items for Systematic Reviews and Meta-Analysis (PRISMA) guidelines [14]. The protocols have been registered in the International Prospective Register of Systematic Reviews (PROSPERO) with registration number CRD42023473209.

### 2.2. Definitions and Diagnosis

Childhood growth hormone deficiency (GHD) is defined by clinical manifestations of having a slow or stagnant rate of growth when compared to peers of the same age. The absence or low level of growth hormone (GH) signifies GHD in a child [4]. Based on the etiology, GHD in children is classified as organic or idiopathic. Diagnosis is established through a combination of factors, including pituitary disease, hypopituitarism, and a reduction in insulin-like growth factor I (IGF-I) concentration or diminished GH responses to various stimuli [15,16]. Based on current guidelines, GH stimulation tests are conducted on children presenting with auxological data indicative of GHD (such as current height < −3 standard deviation (SD) below the mean, current height < −1.5 SD below mid-parental height, and/or current height < −2 SD below the mean combined with a height decrease > 0.5 SD over 1 year in a child older than 2 years). The IGF-1 levels were evaluated against standardized reference ranges for sex and age without substantial disproportionality (sitting height to total height ratio < −2 SD or ≥ +2 SD). Children exhibiting a maximum GH concentration < 10 μg/L in both clonidine and insulin hypoglycemia tests are classified as having GHD, with sex-steroid priming conducted for those aged 9 years and older [17].

### 2.3. Inclusion and Exclusion Criteria

Inclusion and exclusion criteria in this study adopted the PICOS framework (Population, Intervention, Control, Outcome, and Study Design), following the suggestions of previous systematic reviews [18,19]. Included studies had the following criteria: (1) population: prepubertal boys and girls (Tanner stage 1, >2.5 years old) diagnosed with GHD; (2) intervention: a single subcutaneous dose of Somapacitan once-weekly (Sogroya^®^ Novo Nordisk Inc., Plainsboro, NJ, USA); (3) control: daily growth hormone injection or somatropin (Norditropin^®^, SimpleXx^®^, and Nordiflex^®^ [Novo Nordisk Inc., Plainsboro, NJ, USA]); (4) outcome: efficacy, safety, or adherence profile of Somapacitan in children with GHD; and (5) study: randomized controlled trial. Studies recruiting participants with the following conditions were excluded: (1) small gestational babies; and (2) receiving long-acting growth hormones other than Somapacitan. Moreover, studies were excluded if they had one of the following criteria: (1) published in a language other than English; (2) not reporting one of the outcomes of interest; (3) review articles, observational studies, conference abstracts, editorials, commentary, case report, case series, and erratum; and (4) irretrievable full-text articles.

### 2.4. Searching Strategy

A search for the published records was carried out on 17 October 2023 utilizing the searching feature available on PubMed, Embase, and Scopus. Boolean operators (‘OR’/’AND’) were employed in the three databases. The search employed MeSH terms “growth hormone deficiency” AND “somapacitan” OR “sogroya” OR “long-acting growth hormone,” with full keywords presented in Table S1. Results were deduplicated and screened against pre-specified criteria using Zotero version 6.0.30. Bibliographies of included studies were manually screened. Two independent authors (C.A.N. and M.R.P.) screened titles and abstracts retrieved from the searches and assessed these for eligibility against the predetermined inclusion criteria (PICOS). We retrieved all titles and abstracts meeting the inclusion criteria in full text and read these full-text articles to assess eligibility. Disagreements between reviewers were resolved by a third investigator (G.T.) in a blinded manner for discrepancies.

### 2.5. Data Extraction

The included studies focused on primary research investigating the efficacy, safety, and adherence of Somapacitan in treating children. Characteristics, comparisons, and outcomes were collected from the research articles by three independent authors (G.T., C.A.N. and M.R.P.) following pre-established data collection standards. Essential data extracted from eligible full-text articles included the first author’s name, publication year, study sites and countries, trial phase, study period, sample size (intervention and control groups), sample characteristics (gender, age, disease etiology, body mass index), intervention and control doses, means, standard deviations (SDs), and estimated treatment differences (ETD) for intended outcomes at baseline, post-intervention, and changes between the two.

Primary study outcomes included (1) efficacy, measured by height velocity (cm/y), height velocity SDs, height SDs, insulin-like growth factor-1 (IGF-1) SDs, and bone age vs. chronological age ratio; (2) safety, assessed through adverse events and injection site reactions; and (3) adherence, determined by the percentage of the sample completing treatments. Secondary outcomes evaluated disease burden scores, divided into three subgroup domains: emotional well-being, physical functional, and social well-being scores. As suggested by previous meta-analyses [20], unless otherwise specified, data retrieved as median were converted into mean by using a free online calculator (https://www.math.hkbu.edu.hk/~tongt/papers/median2mean.html accessed on 20 October 2023). In addition, BMI was expressed as body mass index-for-age percentiles based on Centers for Disease Control and Prevention of the United States (CDC) criteria considering the difference in clinical implications between 8-year-olds versus 4-year-olds. The nutrition status of the children was classified as underweight for BMI < 5th percentile; healthy weight for BMI ≥ 5th percentile to <85th percentile; overweight for BMI ≥ 85th percentile to <95 percentile; obesity for BMI ≥ 95 percentile; and severe obesity for the BMI ≥ 120% of the 95th percentile or ≥ 35 kg/m^2^ [21]. In the case of missing or insufficient data, the corresponding authors would be contacted.

### 2.6. Risk of Bias Assessment

Two independent authors (P.O.Z. and G.T.) assessed the quality of included studies using the Cochrane risk-of-bias tool for randomized trials (RoB 2.0) [22]. The evaluation covered five domains: randomization bias, bias of intended intervention deviation, missed result data, measurement result, and bias of reported result. The assessment of randomization bias involves evaluating allocation sequence generation and allocation sequence concealment, while the bias of intended intervention deviation domain examines the blinding of participants, carers, healthcare providers, or trial personnel to interventions, along with assessing missed result data due to withdrawn or lost participants and other instances of missing data. Additionally, in the measurement results domain, the awareness of assessors and the likelihood of intervention influence on knowledge are considered, and the probability of biased reports is observed by defining an outcome domain as a true state, irrespective of how it is measured, an outcome encompassing both measurement and outcome analysis. Ratings were categorized as high risk, some concerns, or low risk. Any “high” or “some concerns” rating in a single domain affected the overall study quality categorization. Disagreements were resolved by involving all authors.

### 2.7. Statistical Analysis

Effect sizes were presented as mean differences (MD) and standard error for continuous outcomes and risk ratios (RR) with 95% confidence intervals (95% CI) for dichotomous outcomes. The effect sizes were calculated based on the random-effects model. The random-effects model was used following the consideration of the different locations, populations, and periods of study in each of the studies. Statistical heterogeneity was assessed with *I*^2^ statistics, categorizing heterogeneity as low if *I*^2^ > 50% or *p* < 0.1 [23]. The adherence rate was calculated by multiplying the pooled proportion by 100%. The pooled proportion was obtained from a meta-analysis using Jamovi v.2.3.21 (https://www.jamovi.org/ accessed on 25 October 2023), as suggested by previous studies [24,25]. Pooled estimates were based on the outcome of the Phase II clinical trial. An estimate with a *p*-value < 0.05 was considered statistically significant. Subgroup analyses were conducted based on disease burden parameters (emotional well-being score, physical functional score, and social well-being score), symptom severity (mild, moderate, and severe), being related to trial products (probably related, possibly related, unlikely related), and serious events. Unless otherwise specified, all statistical analyses were performed on RevMan v.5.4 (https://revman.cochrane.org/ accessed on 25 October 2023).

## 3. Results

### 3.1. Searching Result and Study Characteristics

**Figure 1:**
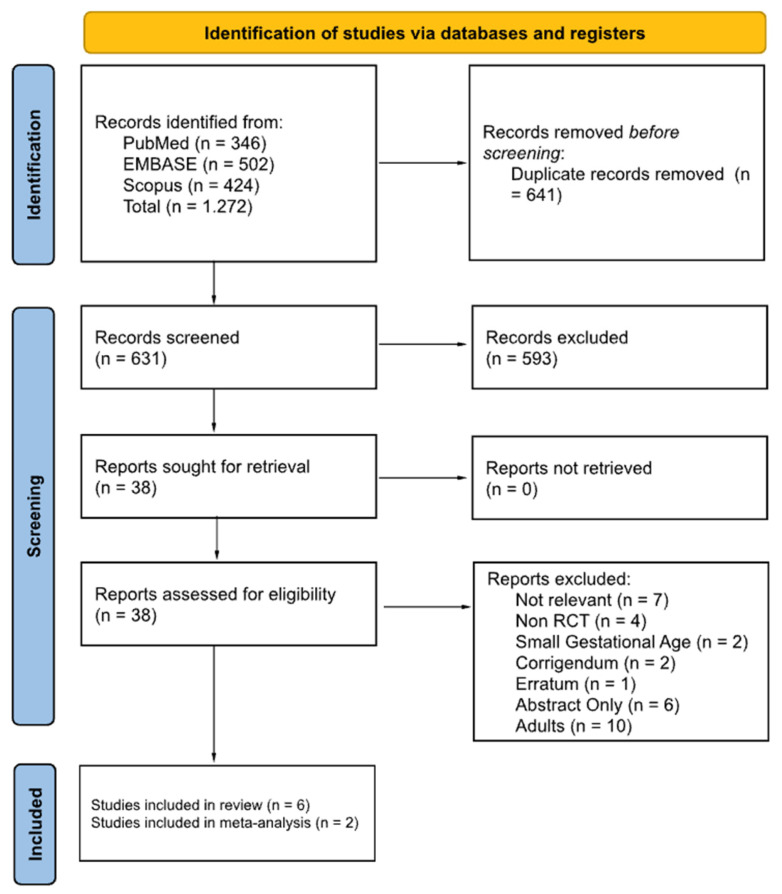
PRISMA flowchart of the included studies.

The literature search yielded 1272 studies, with 6 included in the systematic review and 2 in the meta-analysis. The systematic search and inclusion process was conducted following the PRISMA method, as shown in the flowchart presented in Figure 1. The characteristics of the included studies (published between 2017 and 2023) have been summarized and presented in Table 1. The summary of the subjects’ characteristics, intervention, control, and outcomes is presented in Table 2.

The study included 289 prepubertal children diagnosed with GHD, with 206 males and 83 females. Etiologies were classified as idiopathic in 252 cases and organic in 37 cases. Somapacitan was administered at doses ranging from 0.02 to 0.16 mg/kg/week. The average age of the children was 6.58 ± 2.29 years. Five studies were conducted in various countries across Asia, Europe, North America, and the Middle East, except for the study by Battelino et al. in 2017 which did not specify the countries [11]. The outcomes were measured from the start of administration to the end of observation time. All randomized controlled trials included in this present study utilized a daily growth hormone injection with a control dose of 0.034 mg/kg/day [11,26,27,28,29,30]. The present meta-analysis focuses specifically on the 0.16 mg/kg/week Somapacitan dosage, supported by available data and the prediction of achieving higher insulin-like growth factor-I (IGF-I) levels compared to daily human growth hormones [31].

### 3.2. Quality Assessment

**Figure 2:**
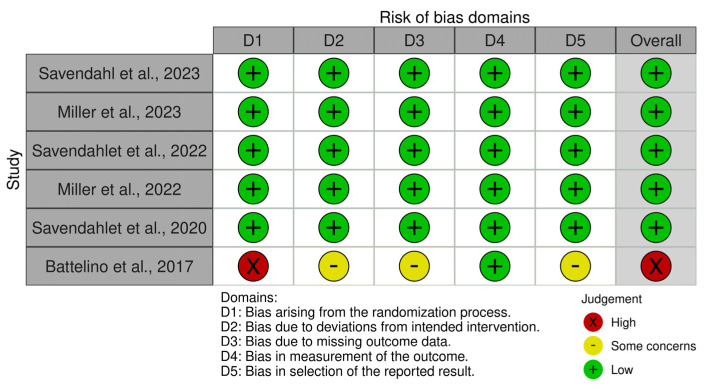
Traffic-light plot for the risk of bias of the included studies according to Cochrane’s RoB 2.0.

Risks of bias from the reporting of the included studies have been analyzed and presented in Figure 2. Overall, the included studies had a low risk of bias (83.3%) and high risk of bias (16.67%). Battelino et al. 2017 received high-risk remarks due to non-blinding intervention and lack of information about the concealment of the allocation sequence and may not be a primary concern in Phase 1 trials, as the emphasis is typically on understanding the basic characteristics of the investigational treatment [11].

### 3.3. Efficacy of Somapacitan Compared to Daily Growth Hormone/Norditropin

**Figure 3:**
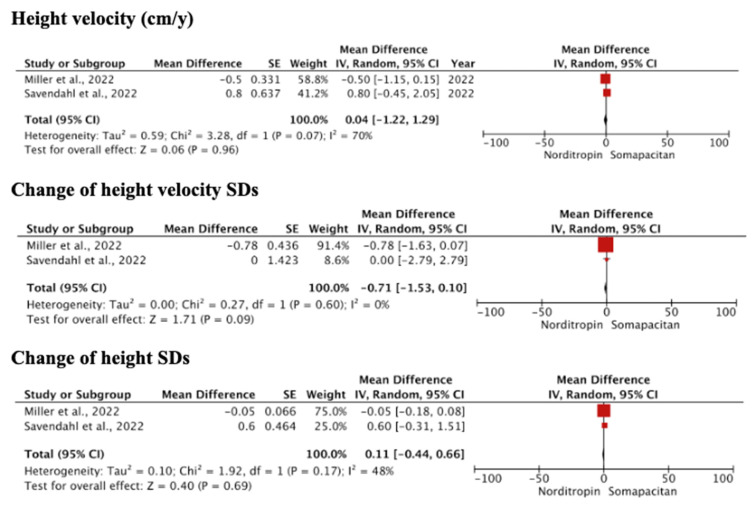

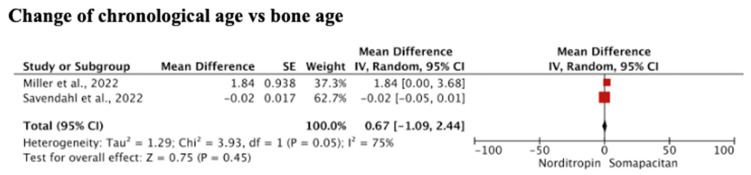
Forest plot of efficacy comparing Somapacitan at a dose of 0.16 mg/kg/week to daily growth hormone/Norditropin at 0.034 mg/kg/day. Size of the red box indicates the study weight. The black diamond indicates the value for the total estimates.

Pooled estimates on the efficacy of Somapacitan as compared with Norditropin are presented in Figure 3. The results revealed similar outcomes in height velocity, height SDs, IGF-I SDs, and ‘chronological age vs. bone age’. Non-inferiority was observed in height velocity SDs (MD = −0.71 (95% CI: −1.53, 0.10); *p* = 0.09). Heterogeneities were found in high velocity and ‘change in chronological age vs. bone age’ pooled estimates (*I*^2^ > 50%, *p* < 0.1). Meanwhile, the pooled estimates of ‘change in height velocity SDs’, ‘change in height SDs’, and ‘change in IGF-I SDs’ were rather non-heterogenous (*I*^2^ < 50%, *p* > 0.1).

### 3.4. Adverse Event of Somapacitan Compared to Daily Growth Hormone/Norditropin

**Figure 4:**
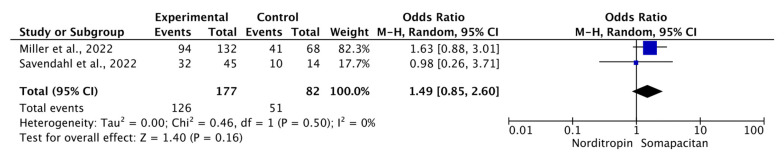
Forest plot of overall adverse events of Somapacitan versus Norditropin. Size of the blue box indicates the study weight. The black diamond indicates the value for the total estimates.

Safety profiles were similar between the two groups, with an overall adverse event OR of 1.49 (95% CI: 0.85, 2.60; *p* = 0.16; *I*^2^ = 0%) (Figure 4). Pooled estimates for subgroups of adverse events are presented in Table 3. Detailed subgroup forest plots for adverse events are presented in Figures S1–S3.

### 3.5. Treatment Adherence of Somapacitan Compared to Daily Growth Hormone/Norditropin

**Figure 5:**
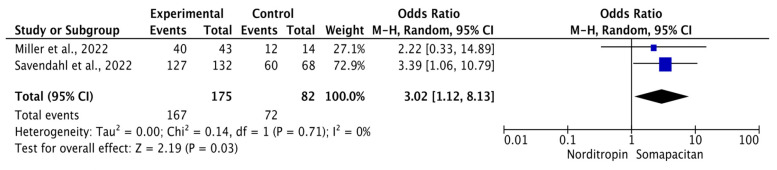
Forest plot of odds ratio adherence in Somapacitan compared to daily growth hormone/Norditropin. Size of the blue box indicates the study weight. The black diamond indicates the value for the total estimates.

Treatment adherence is three times higher in the Somapacitan group compared to Norditropin (OR = 3.02 (95% CI:1.12, 8.13); *p* = 0.03) (Figure 5). The proportion pooled estimates revealed that the adherence rates reached 95% and 88% for Somapacitan and Norditropin with no heterogeneity (*I*^2^ = 0%), respectively (Figures S4 and S5).

### 3.6. Disease Burden

**Figure 6:**
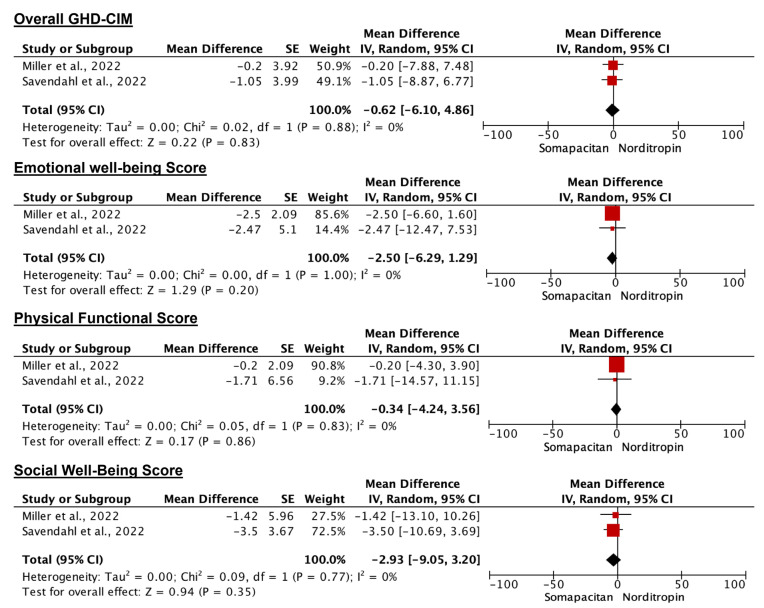
Forest plot for the Growth Hormone Deficiency–Child Impact Measure (GHD-CIM) comparing Somapacitan to daily growth hormone/Norditropin. Size of the red box indicates the study weight. The black diamond indicates the value for the total estimates.

Disease burden measurement is comparable in both groups, as indicated by the Growth Hormone Deficiency–Child Impact Measure (GHD-CIM) which comprises three domains, namely emotional well-being, physical well-being, and social well-being. The pooled estimates for the disease burden are presented in Figure 6**.** For the overall GHD-CIM, the MD was −0.62 (95% CI: −6.10, 4.86) with no statistical significance (*p* = 0.83). Analysis of each domain also yielded no statistical significance (*p* > 0.5). Heterogeneity was not observed in the pooled estimates of GHD-CIM or its three specific domains (*I*^2^ = 0%, *p* > 0.7).

## 4. Discussion

### 4.1. Efficacy, Safety, and Adherence

Several long-acting growth hormone products, such as Eutropin Plus^®^, Jintrolong^®^, Somapacitan (Sogroya^®^), Somatrogon (NGENLA^®^), and Lonapegsomatropin (Skytrofa^®^), have been developed using different approaches to extend growth hormone molecule half-life [13,28]. As Somapacitan has been approved by the FDA for both children and adults, Novo Nordisk is in the process of making Sogroya^®^ available in the coming months [12]. The present study assessed the efficacy, safety, and adherence of Somapacitan versus daily growth hormone in children with GHD, specifically focusing on the dosage of 0.16 mg/kg/week. We found that the efficacy and safety profile of Somapacitan were comparable to daily growth hormones, showing similar and non-inferior outcomes. Nevertheless, treatment adherence is three times higher in the Somapacitan group.

Conflicting results have been presented regarding height velocity between Somapacitan and daily growth hormone injections for children with GHD. Miller et al., (2023) established a non-inferiority threshold of −1.8 cm/year for Somapacitan [26]. In contrast, Savendahl et al., (2020) reported superiority, indicating a significant increase in mean annualized height velocity compared to daily growth hormone injections at week 52 (MD = 1.8 cm/y (95% CI: −0.4.–2.1)) [30]. In the present study, we observed a similar efficacy regarding height velocity between Somapacitan and daily growth hormone injections, underscoring the need for additional research to draw optimal conclusions.

In a Phase I study, a once-weekly dose of 0.16 mg/kg of Somapacitan is predicted to elevate IGF-I levels compared to daily growth hormone injections, staying below +2 SDs on average based on a single injection evaluation [31]. However, the present study found a similar result in IGF-I levels between Somapacitan and Norditropin. This discrepancy may be attributed to the imbalance baseline of the Phase I study that was addressed by adjusting the baseline and potentially introducing bias. It is worth noting that, in the present systematic review, the study was labeled as ‘high-risk’. Furthermore, the interval of the study is 7 weeks, which is a short period to assess all the outcomes. The observed outcome is reasonable as, with long-acting growth hormone administration, serum IGF-I levels tend to reach their highest point shortly after dosing and then gradually decline until the subsequent injection. This pattern contrasts with the consistent and lower IGF-I profile observed in individuals receiving daily growth hormone injections [32].

The molecule of Somapacitan comprises a human growth hormone (GH) protein (22 kDa) with a single amino acid substitution at position 101 (leucine to cysteine). It includes a hydrophilic linker sequence and an albumin-binding moiety (1.2 kDa) that has been attached. This endogenous albumin affects the delay in the elimination of Somapacitan, thereby prolonging the duration of action. This well-established protraction method has been proven to extend half-life (t½) in insulin and glucagon-like peptide-1 [33]. At low doses of Somapacitan, nearly the entire dose is absorbed through the zero-order pathway, gradually entering the central compartment, resulting in plasma concentrations that exhibit standard linear kinetics. Conversely, at high concentrations of Somapacitan, a non-linear shoulder in pharmacokinetics is observed, indicating rapid absorption of a significant fraction [31].

Adverse events observed with Somapacitan are similar to those associated with daily growth hormone, with a prevalence of mild and moderate symptoms, particularly higher mild symptoms in the Somapacitan group. Safety evaluations spanning 2 years and 4 years highlight nasopharyngitis as the most commonly reported adverse event [26,27]. Moreover, injection site reactions, including hematoma, bruising, hemorrhage, swelling, hypersensitivity, macule, mass, hip deformity, and skin atrophy are significant adverse events reported in the studies [26,29]. The long-term safety profile of Somapacitan is under continuous research, focusing on potential risks like tumor recurrence and malignant tumor development. Vigilant monitoring is crucial due to recombinant human growth hormone’s (rhGH) role in cell growth pathways, emphasized by Boguszewski. Being the first long-acting growth hormone (LAGH) with efficacy comparable to rhGH, Somapacitan requires ongoing observation for safety, including cancer risk [34]. Evaluation of the risk of tumor recurrence by employing magnetic resonance imaging is required, especially among individuals with a history of pituitary tumors.

The phenomenon of missing daily growth hormone doses is associated with the fear of needles, leading to reduced efficacy [32]. Long-acting growth hormone appears to address this issue by requiring fewer injections, providing increased acceptance and therapeutic flexibility for both children and adults. Supported by our findings, which show that Somapacitan has significantly three times higher adherence than Norditropin, most parents and caretakers (81.8%) strongly prefer Somapacitan over daily growth hormone injections [27].

The Growth Hormone Deficiency–Child Impact Measure (GHD-CIM) assesses the disease impact and quality of life improvement in children with GHD [35]. Lower scores indicate better health and greater burden reduction. Both Somapacitan and daily growth hormone injection show similar reductions in disease burden, aligning with comparable efficacy outcomes. Two studies reported reduced GHD-CIM scores across all domains (social well-being, physical well-being, and social well-being), attributed to the lower injection frequency of once-weekly Somapacitan treatment [27,28].

### 4.2. Limitations

We did not manually search for the unpublished reports by contacting renowned experts, which could contribute to the selection bias. Moreover, the selection of bias could be derived from our absence in searching the literature from the grey database such as Google Scholar. However, consequently, data included in the present systematic review were derived from peer-reviewed reports implying the high quality of the data. Meta-analysis performed in the present study only included two studies with limited sample size. Moreover, we could not identify the source of heterogeneity in pooled estimates of chronological age vs. bone age and severe adverse events. An updated meta-analysis is warranted to enhance the adequacy and reliability of the overall conclusions following the new findings reported in the future.

### 4.3. Recommendation

For future research, we suggest employing IGF-1 and insulin-like growth factor-binding-3 (IGFBP-3) ratio (IGF-1/IGFBP-3) as a robust parameter to measure the outcome. As reported by a recently published report, the serum IGF-1/IGFBP-3 ratio serves as an accurate marker for diagnosing GDH in children without being affected by concurrent disorders [36]. We also encourage clinical trials in different settings and populations considering their effect on growth patterns and growth disorders. Moreover, the GDH status is also influenced by nutrition intake and living conditions, highlighting the importance of recruiting participants from different economic backgrounds. Since the included studies herein mostly recruited higher numbers of male participants, recruitment of female participants is encouraged for future research since gender underlies the difference in growth patterns and growth disorders.

## 5. Conclusions

Although the efficacy and safety profiles of Somapacitan were found to be similar to those of daily growth hormone, a reduced frequency of once-weekly Somapacitan injections led to increased adherence and can affect the improvement of disease. Based on the data from a study involving a total of 289 prepubertal children, and showing negligible heterogeneity for almost all outcomes, the level of confidence is suggested to be strong. In the case of chronological age vs. bone age and the subgroup of severe adverse events, where the heterogeneity reached more than 50% suggesting a moderate level of evidence. Regardless of its efficacy, Somapacitan should be prescribed with caution for the risk of tumor recurrence, especially among individuals with a history of pituitary tumors. Future trials with a larger participant pool are warranted to comprehensively evaluate Somapacitan.

## Figures and Tables

**Table 1:** Characteristics of the included studies.

Study, Year (Ref)	Sites/Country (n)	Observation Time *	Sample Size (I/C)	Trial Phase (Number)
Miller et al., 2023 [26]	86/20	104 weeks	200 (132/68)	III (NCT03811535)
Savendahl et al., 2023 [27]	29/11	208 weeks	57 (43/14)	III (NCT02616562)
Miller et al., 2022 [28]	86/20	52 weeks	200 (132/68)	II (NCT03811535)
Savendahl et al., 2022 [29]	29/11	156 weeks	57 (43/14)	II (NCT02616562)
Savendahl et al., 2020 [30]	29/11	52 weeks	57 (43/14)	II (NCT02616562)
Battelino et al., 2017 [11]	14/8	7 days	32 (24/8)	I (NCT01973244)

* Acts as endpoints for outcome measurement.

**Table 2:** Characteristics of subjects, intervention, and outcomes.

Study (Year) [Ref]	Subjects’ Characteristics				Somapacitan^®^ Dose (mg/kg/week)	Control	**Outcomes**	**Injection Site Reaction (I/C)**
	Etiology of GHD, I/O	Sex, M/F	Age, Mean ± SD (Years)	BMI (kg/m^2^) [Percentile, Status]				
Miller et al., 2023 [26]	176/24	149/51	6.4 ± 2.3	15.7 ± 1.5 (<1st, underweight)	0.16	Norditropin^®^ (0.034 mg/kg/d)	HV (cm/y), HV SDs, height SDs, IGF-1 SDs, BA vs. CA.	NR
Savendahl et al., 2023 [27]	53/4	34/23	5.95 ± 1.98	15.14 ± 1.20 (<1st, underweight)	0.16	Norditropin^®^ (0.034 mg/kg/d)	HV (cm/y), HV SDs, height SDs, IGF-I SDS, IGFBP-3 SDs, GHD-CTB, GHD-PTB.	NR
Miller et al., 2022 [28]	176/24	149/51	6.4 ± 2.3	15.7 ± 1.52 (<1st, underweight)	0.16	Norditropin^®^ (0.034 mg/kg/d)	HV (cm/y), HV SDs, height SDs, IGF-1 SDs, BA vs. CA, GHD-CIM, GHD-CTB	7/4
Savendahl et al., 2022 [29]	53/4	34/23	5.95 ± 1.98	15.14 ± 1.20 (<1st, underweight)	0.16	Norditropin^®^ (0.034 mg/kg/d)	HV (cm/y), HV SDs, height SDs, IGF-I SDs, IGFBP-3 SDs, bone age, GHD-CIM	NR
Savendahl et al., 2020 [30]	53/4	34/23	5.95 ± 1.98	15.14 ± 1.20 (<1st, underweight)	0.04, 0.08, or 0.16	Norditropin^®^ (0.034 mg/kg/d)	HV (cm/y), HV SDs, height SDs, IGF-I SDs, IGFBP-3 SDs, bone age, GHD-CIM, HbA1c	3/1
Battelino et al., 2017 [11]	23/9	23/9	8.85 ± 1.30	16.16 ± 2.48 (<1st, underweight)	0.02, 0.04, 0.08, or 0.16	Norditropin^®^ (0.034 mg/kg/d)	Cmax, tmax, IGF-I level, IGFBP-3 level, IGF-1 SDs, IGFBP-3 SDs	4/0

Abbreviations: BA, bone age; CA, chronological age; Cmax, peak plasma concentration; GHD, growth hormone deficiency; GHD-CIM, growth hormone deficiency–child impact measure; GHD-CTB, growth hormone deficiency–child treatment burden; GHD-PTB, growth hormone deficiency–parent treatment burden; HV, high velocity; IGF-1, insulin-like growth factor-1; I/C, intervention/control; I/O, idiopathic/organic; IGFBP-3, insulin-like growth factor-binding-3; M/F, male/female; NR, not reported; SDs, standard deviation score; tmax, time to Cmax; and BMI (kg/m^2^) expressed in mean ± SD.

**Table 3:** Summary of the overall effects for subgroups of adverse events.

Subgroups	Events with Somapacitan	Events with Norditropin	OR (95% CI)	*p*-Value	*I*^2^ (%)
Severity					
Mild symptoms	117/175	35/82	1.66 (0.97, 2.85)	0.06	0%
Moderate symptoms	38/175	14/82	1.30 (0.66, 2.59)	0.45	0%
Severe events	4/175	2/82	0.58 (0.03, 11.14)	0.72	57%
Relation to trial products					
Probably related	16/175	5/82	1.52 (0.54, 4.32)	0.43	0%
Possibly related	24/175	11/82	1.03 (0.48, 2.23)	0.93	0%
Unlikely related	122/175	48/82	1.54 (0.89, 2.67)	0.12	0%
Serious event					
Serious event	10/175	4/82	1.01 (0.30, 3.40)	0.98	0%

## Data Availability

All underlying data have been presented in this article.

## Supplementary Materials

The following supporting information can be downloaded at https://www.mdpi.com/article/10.3390/children11020227/s1, Figure S1: forest plot of subgroup odds ratio of adverse events symptoms; Figure S2: adverse events related to trial products; Figure S3: serious adverse events; Figure S4: forest plot of proportions of adherence of Somapacitan; Figure S5: forest plot of proportions of adherence of Norditropin; Table S1: detailed keywords used in databases.
