# Supplementary material for: Once-Weekly Somapacitan as an Alternative Management of Growth Hormone Deficiency in Prepubertal Children: A Systematic Review and Meta-Analysis of Randomized Controlled Trial
Source: Children (Basel). 2024 Feb 9;11(2):227. doi: 10.3390/children11020227 (PMC10887308; doi:10.3390/children11020227)
Supplement: Supplementary file 1 [file children-11-00227-s001.zip › children-2812243-supplementary.pdf]

children

Supplementary Materials

Table S1. Detailed keywords used in databases.

Database	Keyword combination
PubMed	1. (long-acting OR "long acting" OR weekly OR once-weekly) AND (growth hormone OR gh OR hgh OR rhgh OR somatropin OR somatotropin)<br>2. LAGH<br>3. Sogroya<br>4. somapacitan<br>5. #1 OR #2 OR #3 OR #4<br>6. Growth Hormone Deficiency OR GH Deficiency<br>7. #5 AND #6
EMBASE	1. (long-acting OR "long acting" OR weekly OR once-weekly) AND (growth hormone OR gh OR hgh OR rhgh OR somatropin OR somatotropin)<br>2. LAGH OR Sogroya OR somapacitan<br>3. #1 OR #2<br>4. Growth Hormone Deficiency OR GH Deficiency<br>5. #3 AND #4
Scopus	1. Sogroya<br>2. somapacitan<br>3. LAGH<br>4. ((long-acting OR "long acting" OR weekly OR once-weekly) AND (growth hormone OR gh OR hgh OR rhgh OR somatropin OR somatotropin)<br>5. #1 OR #2 OR #3 OR #4<br>6. Growth Hormone Deficiency OR GH Deficiency<br>7. #5 AND #6

Study or Subgroup	Experimental Events	Experimental Total	Control Events	Control Total	Weight	Odds Ratio M-H, Random, 95% CI
**4.1.1 Mild Symptomps**						
Miller et al., 2022	87	132	35	68	47.9%	1.82 [1.00, 3.31]
Savendahl et al., 2022	30	45	9	14	10.8%	1.11 [0.32, 3.90]
**Subtotal (95% CI)**		**177**		**82**	**58.6%**	**1.66 [0.97, 2.85]**
Total events	117		44			
Heterogeneity: Tau² = 0.00; Chi² = 0.49, df = 1 (P = 0.49); I² = 0%						
Test for overall effect: Z = 1.85 (P = 0.06)						
**4.1.2 Moderate Symtomps**						
Miller et al., 2022	27	132	12	68	30.0%	1.20 [0.56, 2.55]
Savendahl et al., 2022	11	45	2	14	6.3%	1.94 [0.38, 10.05]
**Subtotal (95% CI)**		**177**		**82**	**36.3%**	**1.30 [0.66, 2.59]**
Total events	38		14			
Heterogeneity: Tau² = 0.00; Chi² = 0.27, df = 1 (P = 0.60); I² = 0%						
Test for overall effect: Z = 0.76 (P = 0.45)						
**4.1.3 Severe Events**						
Miller et al., 2022	4	132	1	68	3.5%	2.09 [0.23, 19.11]
Savendahl et al., 2022	0	45	1	14	1.6%	0.10 [0.00, 2.57]
**Subtotal (95% CI)**		**177**		**82**	**5.1%**	**0.58 [0.03, 11.14]**
Total events	4		2			
Heterogeneity: Tau² = 2.64; Chi² = 2.31, df = 1 (P = 0.13); I² = 57%						
Test for overall effect: Z = 0.36 (P = 0.72)						
**Total (95% CI)**		**531**		**246**	**100.0%**	**1.47 [0.97, 2.22]**
Total events	159		60			
Heterogeneity: Tau² = 0.00; Chi² = 3.82, df = 5 (P = 0.58); I² = 0%						
Test for overall effect: Z = 1.82 (P = 0.07)						
Test for subgroup differences: Chi² = 0.70, df = 2 (P = 0.71), I² = 0%						

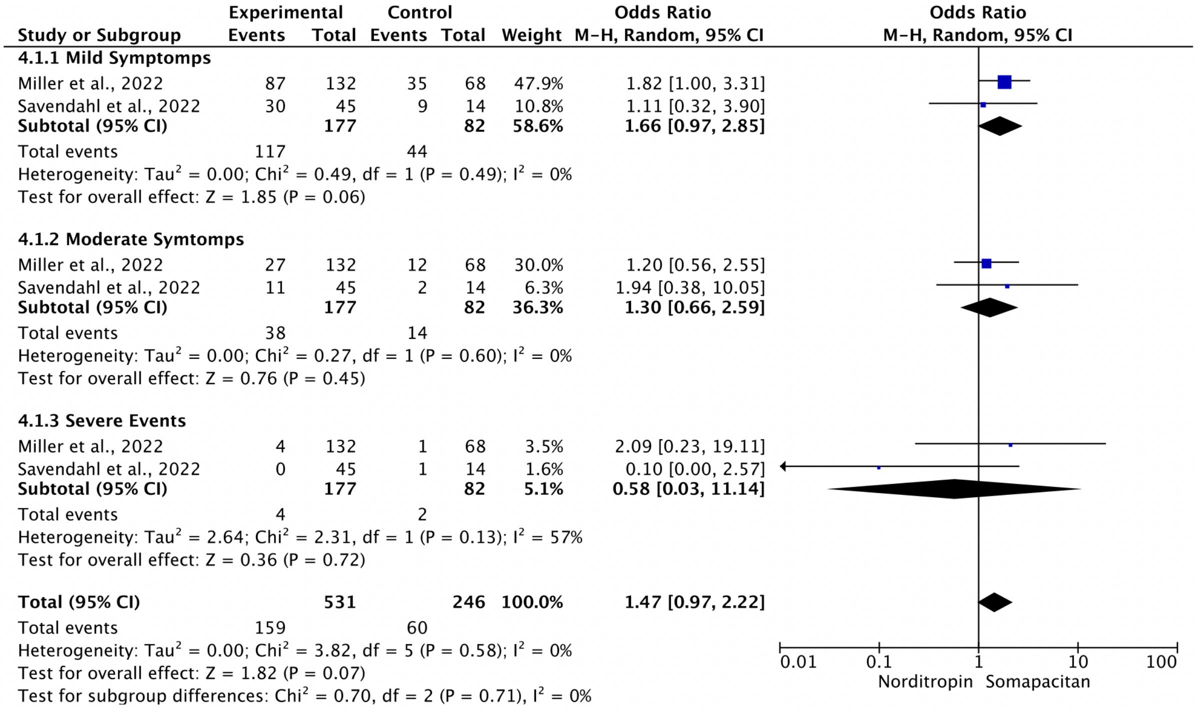

Figure S1. Forest plot of subgroup odds ratio of adverse events symtomps.

Study or Subgroup	Experimental Events	Experimental Total	Control Events	Control Total	Weight	Odds Ratio M-H, Random, 95% CI
**4.2.1 Probably related**						
Miller et al., 2022	12	132	4	68	12.3%	1.60 [0.50, 5.16]
Savendahl et al., 2022	4	45	1	14	3.2%	1.27 [0.13, 12.38]
**Subtotal (95% CI)**		**177**		**82**	**15.5%**	**1.52 [0.54, 4.32]**
Total events	16		5			
Heterogeneity: Tau² = 0.00; Chi² = 0.03, df = 1 (P = 0.86); I² = 0%						
Test for overall effect: Z = 0.79 (P = 0.43)						
**4.2.2 Possibly related**						
Miller et al., 2022	20	132	9	68	23.4%	1.17 [0.50, 2.73]
Savendahl et al., 2022	4	45	2	14	5.1%	0.59 [0.10, 3.60]
**Subtotal (95% CI)**		**177**		**82**	**28.6%**	**1.03 [0.48, 2.23]**
Total events	24		11			
Heterogeneity: Tau² = 0.00; Chi² = 0.46, df = 1 (P = 0.50); I² = 0%						
Test for overall effect: Z = 0.09 (P = 0.93)						
**4.2.3 Unlikely related**						
Miller et al., 2022	90	132	38	68	46.4%	1.69 [0.93, 3.09]
Savendahl et al., 2022	32	45	10	14	9.6%	0.98 [0.26, 3.71]
**Subtotal (95% CI)**		**177**		**82**	**55.9%**	**1.54 [0.89, 2.67]**
Total events	122		48			
Heterogeneity: Tau² = 0.00; Chi² = 0.53, df = 1 (P = 0.47); I² = 0%						
Test for overall effect: Z = 1.55 (P = 0.12)						
**Total (95% CI)**		**531**		**246**	**100.0%**	**1.37 [0.91, 2.07]**
Total events	162		64			
Heterogeneity: Tau² = 0.00; Chi² = 1.76, df = 5 (P = 0.88); I² = 0%						
Test for overall effect: Z = 1.51 (P = 0.13)						
Test for subgroup differences: Chi² = 0.73, df = 2 (P = 0.69), I² = 0%						

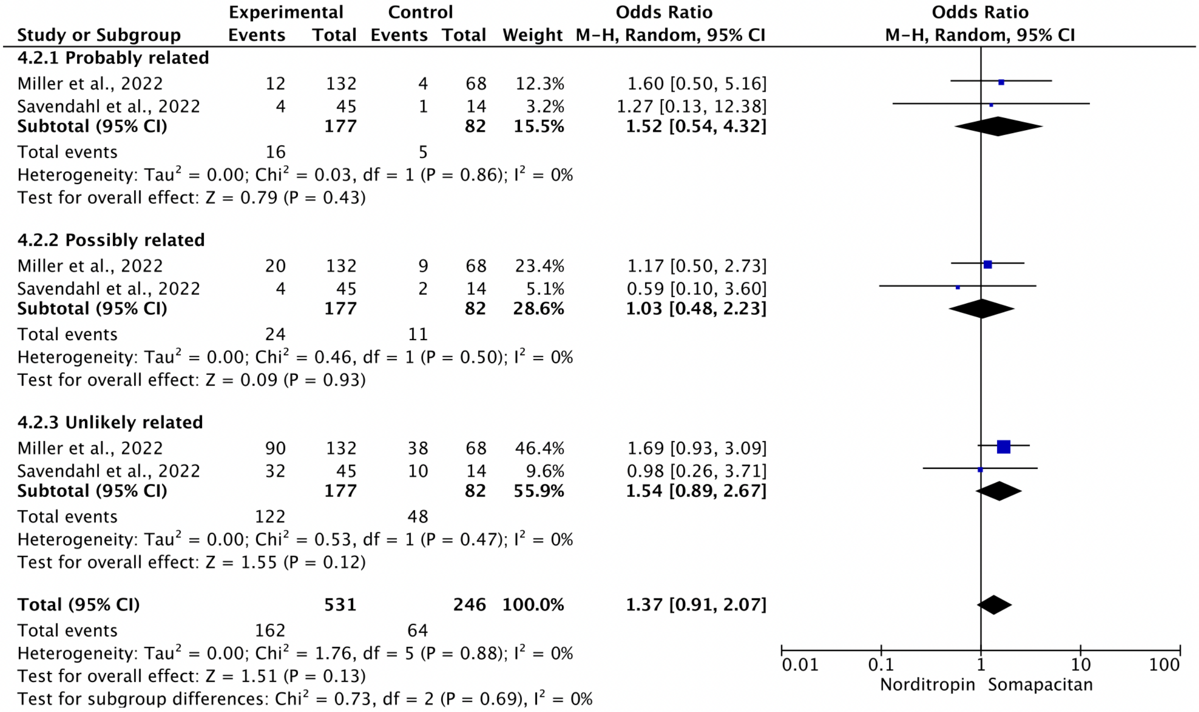

Figure S2. Adverse events related to trial products.

Study or Subgroup	Experimental Events	Experimental Total	Control Events	Control Total	Weight	Odds Ratio M-H, Random, 95% CI
Miller et al., 2022	4	45	2	14	44.6%	0.59 [0.10, 3.60]
Savendahl et al., 2022	6	132	2	68	55.4%	1.57 [0.31, 8.00]
**Total (95% CI)**		**177**		**82**	**100.0%**	**1.01 [0.30, 3.40]**
Total events	10		4			

Heterogeneity: Tau^2 = 0.00; Chi^2 = 0.63, df = 1 (P = 0.43); I^2 = 0%
Test for overall effect: Z = 0.02 (P = 0.98)

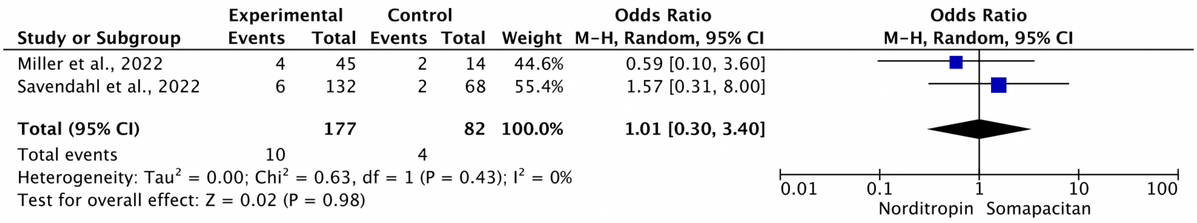

Figure S3. Serious adverse events.

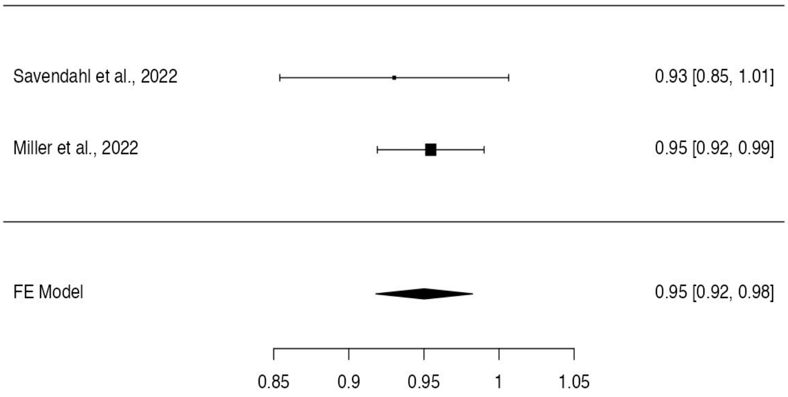

Figure S4. Forest plot of proportions of adherence of Somapacitan.

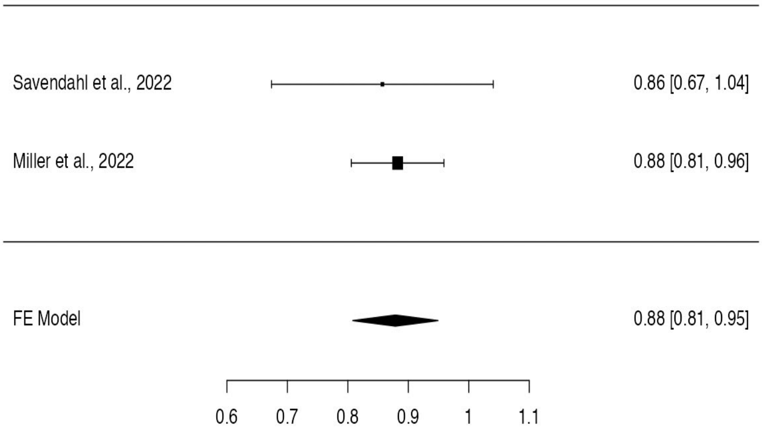

Figure S5. Forest plot of proportions of adherence of Norditropin.
